# Identification and Screening of LITAF Family Key Genes Responsive to Plant Secondary Metabolites in *Helicoverpa armigera*

**DOI:** 10.3390/biology15080595

**Published:** 2026-04-09

**Authors:** Jie Zhao, Xinxin Jin, Haoran Kan, Jing Ye

**Affiliations:** 1 Key Laboratory of Xinjiang for Oasis Agricultural Pests Management and Plant Protection Resources Utilization, Agricultural College, Shihezi University, Shihezi 832003, Chinakanhaoran@stu.shzu.edu.cn (H.K.); 2 Laboratory of Forestry Department, College of Urban and Environmental Sciences, Shihezi University, Shihezi 832003, China

**Keywords:** LITAF, plant secondary metabolite, expression analysis, *Helicoverpa armigera*

## Abstract

Lipopolysaccharide-induced tumor necrosis factor alpha factor (LITAF) as a crucial cytokine can mediate the inflammatory and immune regulatory responses of an organism. It plays a significant mediatory role in the innate immune system of insects. Firstly, the LITAF family of cotton bollworm (*Helicoverpa armigera*, Lepidoptera: Noctuidae) was analyzed, and the differentially expressed genes of *HaLITAFs* were screened from the 2-tridecanone-treated transcriptome. Subsequently, these two differentially expressed *HaLITAF* genes were cloned and analyzed, and the effect of different larval stages, tissues, and plant secondary metabolites on their gene expression were detected in *H. armigera*. The cotton bollworm contains 13 *LITAF* genes, and there are no repetitive sequences among them. Upon 2-tridecanone treatment, only *HaLITAF5* and *HaLITAF7* were significantly upregulated in the LITAF family of cotton bollworm larvae after 6 h. The *Ha*LITAF5 and *Ha*LITAF7 proteins, respectively, comprised 78 and 113 amino acids, and both contained a CXXC motif, hydrophobic amino acid region and HXCPXCXXXXG motif. Both of them belong to the LITAF analogues (zf-LITAF-like) superfamily. The expressions of *HaLITAF5* and *HaLITAF7* were both the lowest in fourth-instar larvae and the highest in sixth-instar larvae, and both were abundantly expressed in the midgut of sixth-instar larvae. Following a 28 h treatment with 2-tridecanone, the expression levels of *HaLITAF5* and *HaLITAF7* peaked. *HaLITAF7* exhibited the highest expression level after a 4 h exposure to tannic acid, whereas *HaLITAF5* reached its peak after 28 h of tannic acid treatment. The maximum expression levels of *HaLITAF5* and *HaLITAF7* were observed after 28 h and 4 h of quercetin treatment, respectively. Subsequent to ZQ-8 treatment, *HaLITAF5* expression peaked at 28 h, while *HaLITAF7* expression peaked at 8 h. Both *HaLITAF5* and *HaLITAF7* were overexpressed after different kinds of plant secondary metabolite stresses, and thereby, they may be regulated in the expression of genes related to downstream detoxification metabolic processes. This provides a theoretical foundation for further studies on the functional mechanism of the *LITAF* gene in *H. armigera*.

## Simple Summary

The cotton bollworm (*Helicoverpa armigera*), a globally significant agricultural pest, displays notable host adaptability and insecticide resistance. This article mainly identified the differentially expressed *LITAF* genes of cotton bollworm in response to plant secondary metabolite stimulus. There were 13 *LITAF* genes in cotton bollworm, and all of them could be regulated by transcription factors that respond to stimulus. Following this, the differentially expressed *HaLITAF5* and *HaLITAF7* genes were identified from the midgut transcriptome after treatment with the plant secondary metabolite 2-tridecanone. The *HaLITAF5* and *HaLITAF7* genes were overexpressed in response to four different plant secondary metabolites. This article has established the groundwork for future investigations into the roles of LITAF in the immune response and detoxification metabolism of cotton bollworm.

## 1. Introduction

Lipopolysaccharide (LPS) induces monocytes and macrophages to secrete tumor necrosis factor alpha (TNF-α) in animals [1]. TNF-α, as a proinflammatory cytokine, could further mediate the inflammatory and immunomodulatory responses of the body and even directly cause tumor cell death [2,3]. In 1997, 14 *p53*-inducible genes (*PIGs*) were screened with significantly upregulated expression in DLD-1, a *p53*-deficient human colon cell line [4]. In 1999, a new protein was isolated from THP-1 with LPS stimulation from a human peripheral blood mononuclear cell line, and this was named LPS-induced TNF-α (LITAF). Then, the cDNA sequence of *LITAF* was sequenced, and it was found that the gene shared 98% homology with *PIG7*, i.e., *LITAF* was *PIG7* [5]. In 2001, another splicing mode of the *PIG7* gene was found in the lysosome of *Mycobacterium bovis*, and this was named small integral membrane protein of the lysosome or late endosomes (SIMPLE) [6]. The distribution of LITAF is widespread, spanning from rotifers to humans [7,8,9]. Despite variations in LITAF sequences among different organisms, they consistently feature a LITAF domain at their C-terminus, also referred to as the SIMPLE-like domain (SLD). This domain is identified by the interruption of the CXXC and HXCXXC motifs with a hydrophobic stretch region and is classified as part of the zinc ribbon family for its zinc-finger domain [10,11]. Research has demonstrated that LITAF can induce the inflammatory response in immune cells, participate in the protein sorting pathway of endocytic vesicles, and even inhibit the occurrence and progression of tumor cells [12,13,14,15].

The cotton bollworm, *Helicoverpa armigera* (Lepidoptera: Noctuidae), is a cosmopolitan and polyphagous pest. Its larvae are capable of feeding on the young leaves, flower buds, and fruits of over 200 plant species across more than 30 families [16]. Throughout plant growth and development, various secondary metabolites are produced, including phenols, terpenoids, and alkaloids. These compounds can inhibit insect feeding, disrupt food digestion and utilization, and may even result in insect poisoning and mortality [17]. Conversely, insects possess a highly efficient detoxification enzyme system capable of metabolizing diverse plant secondary metabolites, facilitating a co-evolutionary relationship between insects and host plants. Key enzymes involved include cytochrome P450 monooxygenases (CYP450s), glutathione S-transferases (GSTs), and carboxylesterases (CarEs) [18]. CYP6B6 serves as a critical detoxifying enzyme in the midgut of *H. armigera*. Its expression can be upregulated in response to various plant secondary metabolites [19,20,21]. Silencing the *CYP6B6* gene also could impede larval growth, disrupt molting and metamorphosis, and even result in larval mortality or adult deformities [22,23]. Thus, CYP6B6 influences both detoxification and growth regulation in *H. armigera*.

Previous studies from our team have reported some regulators of *CYP6B6* gene under 2-tridecanone treatment; they could increase *CYP6B6* expression in *H. armigera* [24,25,26,27]. Furthermore, during the screening of regulators for the *CYP6B6* promoter using a yeast one-hybrid technique, it was discovered that a LITAF protein could bind to its xenobiotic response element, which was *Ha*LITAF5 (data not published). We hypothesize that LITAF may modulate *CYP6B6* expression, influencing detoxification and growth of *H. armigera*. Although insect LITAF reports are limited, multiple *LITAF* genes were identified as overexpressed post-exposure to bacteria or plasmodium in *Drosophila melanogaster* and *Anopheles gambiae* [28,29]. Determining the number of *LITAF* genes in cotton bollworm and identifying those responsive to plant secondary metabolite stress is crucial for elucidating LITAF’s role. Thus, this study investigated the *LITAF* gene family in *H. armigera*. By subjecting the insects to 2-tridecanone stress, two midgut-specific *HaLITAF* genes, *HaLITAF5* and *HaLITAF7*, were identified. The study assessed the expression of *HaLITAF5* and *HaLITAF7* in different larval instars and various tissues of sixth-instar larvae. Additionally, it examined the changes in gene expression levels following exposure to four different plant secondary metabolites. These findings establish the groundwork for future investigations into the roles of LITAF in the immune response and detoxification metabolism of cotton bollworm.

## 2. Materials and Methods

### 2.1. Insect Rearing

The pupae of cotton bollworm were purchased from Keyun Biology (Baiyun Industry Co., Ltd., Jiyuan, China). Rearing methods and culture conditions followed Zhao [30].

### 2.2. Bioinformatic Analysis of LITAF Gene of H. armigera

Genomes of *H. armigera* (assembly accession: GCF_030705265.1), *Bombyx mori* (silkworm) (assembly accession: GCF_030269925.1), and *Drosophila melanogaster* (fruit fly) (assembly accession: GCF_000001215.4) were downloaded from the NCBI database (downloaded on 13 January 2025). The collinearity and conserved domain architecture of the LITAF CDS region in *H. armigera* were analyzed using TBtools-II v2.423 and SMART (https://smart.embl.de, accessed on 19 February 2025), respectively. Relationships among members of the *Ha*LITAF family were inferred by the neighbor-joining method implemented in MEGA 11.

We retrieved LITAF sequences of *Caenorhabditis elegans* (nematode), *Tribolium castaneum* (red flour beetle), *Anopheles gambiae* (mosquito), *Aedes aegypti* (yellow fever mosquito), *Penaeus vannamei* (Pacific white shrimp), *Magallana gigas* (Pacific oyster), *Danio rerio* (zebrafish), *Mus musculus* (house mouse), *Rattus norvegicus* (Norway rat), and *Homo sapiens* (human) from the NCBI database (downloaded on 23 May 2025). Phylogenetic analysis of the *HaLITAF* gene was performed using the maximum likelihood method using MEGA 11.

The 2000 bp promoter sequence upstream of the CDS of each *HaLITAF* gene was extracted from the cotton bollworm genome. Cis-acting elements within these promoters were identified using transcription factor (TF) binding profiles from the Insecta group of JASPAR^2025^ CORE, applying a relative profile score threshold of 95% (scanned on 28 December 2025). From the resulting TF list, we selected those annotated under the Gene Ontology Biological Process term response to stimulus (GO: 0050896). The cis-element arrangements for each *HaLITAF* gene were schematized in SnapGene 6.0.2. Finally, the classification heatmap of corresponding transcription factors for *HaLITAF* genes were generated using TBtools-II v2.423.

### 2.3. Differential Expression Analysis of the LITAF Gene in H. armigera

Sixth-instar larvae of cotton bollworm were fed an artificial diet containing 1% (*w*/*w*) 2-tridecanone, and midgut tissues were collected for transcriptome sequencing. The details of the experimental procedures and transcriptome data followed Zhang [31]. Previous studies indicated that the expression level of *CYP6B6* significantly increases within 20 h following treatment with 1% 2-tridecanone [24]. Consequently, transcriptome sequencing was conducted at two time points: 6 h and 15 h post-treatment. This approach aimed to analyze the factors that may regulate the upregulation of *CYP6B6* expression. Differential expression of *HaLITAF* genes was analyzed from these transcriptomic data of 2-tridecanone treated 6 h and 15 h. Separately, sixth-instar larvae were ground in liquid nitrogen, and total RNA was extracted using a MiniBEST Universal RNA Extraction Kit (Takara Biomedical Technology, Dalian, China) according to the manufacturer’s protocol. First-strand cDNA was synthesized from the RNA with EasyScript First-Strand cDNA Synthesis SuperMix (TRANGEN BIOTECH, Beijing, China).

### 2.4. The Cloning and Sequence Analysis of HaLITAF5 and HaLITAF7

Based on the predicted *HaLITAF5* and *HaLITAF7* sequences in the NCBI database, the specific primers required for CDS, 5′UTR and 3′UTR amplification were designed (Table 1), and PCR amplification was carried out using the cDNA of sixth-instar larvae as a template. The reaction system and cycling conditions for CDS amplification followed the protocol of the Premix Ex Taq Version 2.0 (Takara Biomedical Technology, Dalian, China), while those for RACE PCR adhered to the SMARTer RACE 5′/3′ Kit (Takara Biomedical Technology, Dalian, China). Purified PCR products were ligated into the pMD18-T vector (Takara Biomedical Technology, Dalian, China) and transformed into *Escherichia coli* DH5α-competent cells (TRANGEN BIOTECH, Beijing, China). Positive clones identified by colony PCR were selected for sequencing. The CDS, 5′UTR and 3′UTR sequences were assembled to obtain the full-length cDNA sequences of *HaLITAF5* and *HaLITAF7* using DNAMAN 8.

The open reading frames (ORFs) were predicted using the ORF Finder tool from the NCBI database. Physicochemical parameter analysis and hydrophobicity/hydrophilicity profiling were conducted using the ProtParam (https://www.expasy.org/resources/protparam, accessed on 19 February 2025) and ProtScale (https://www.expasy.org/resources/protscale, accessed on 19 February 2025) tools, respectively. Signal peptides, transmembrane domains, and subcellular localization were predicted using the SignalP (https://services.healthtech.dtu.dk/services/SignalP-6.0/, accessed on 19 February 2025), TargetP (https://services.healthtech.dtu.dk/services/TargetP-2.0/, accessed on 19 February 2025), and TMHMM (https://services.healthtech.dtu.dk/services/TMHMM-2.0/, accessed on 19 February 2025) tools, respectively. Secondary structures of the proteins were predicted via the PredictProtein (https://status.predictprotein.org/, accessed on 19 February 2025), while tertiary structures were modeled using the SWISS-MODEL (https://www.expasy.org/resources/swiss-model, accessed on 19 February 2025) and the PDB database. All protein structure analyses were carried out from 2 to 5 April 2025. Amino acid sequences of *HaLITAF5* and *HaLITAF7* were aligned with reported and immunity-related LITAF sequences from other species using the Clustal X2 software (sequences downloaded on 23 May 2025).

### 2.5. Expression Profiling Analysis of HaLITAF5 and HaLITAF7

Quantitative polymerase chain reaction (qPCR) was used to quantify *HaLITAF5* and *HaLITAF7* expression: across first- to sixth-instar larvae, among tissues of sixth-instar larvae, and in sixth-instar larvae exposed to four plant secondary metabolites (tannic acid, 2-tridecanone, quercetin and ZQ-8). For developmental comparisons, first-instar larvae served as the control; for tissue comparisons, the head was the control; and for each substance stress treatment, the corresponding blank group was the control. Rearing procedures, reference gene selection, qPCR assay and data analysis methods followed Zhao [30].

## 3. Results

### 3.1. Phylogenetic Analysis of HaLITAFs

**Figure 1:**
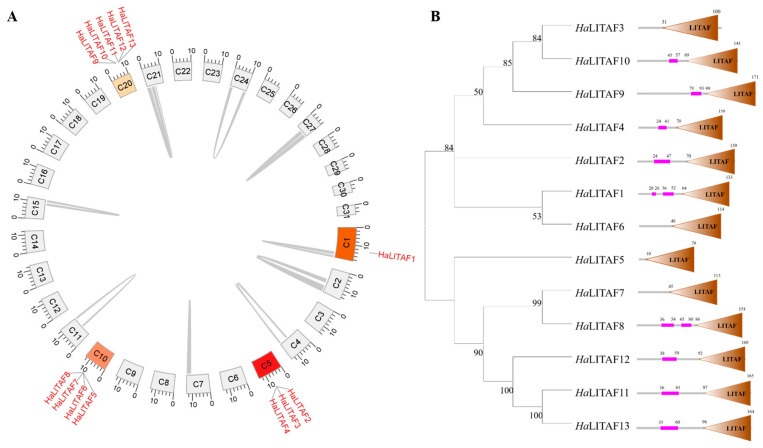
The collinearity and evolutionary relationships of the *HaLITAF* genes. (**A**) The circos plot of the *H. armigera* genome. Chromosomes are represented as circular segments labelled C1 to C31, and the numbers positioned outside the circle indicate the lengths of the chromosomes. Gray lines connecting the circles illustrate the associations between various chromosomal regions. The loci of the *HaLITAF* gene are denoted by red letters, which correspond to their specific locations on the chromosomes. (**B**) The phylogenetic tree and domain pattern diagram of *Ha*LITAF1–*Ha*LITAF13 within the *Ha*LITAF family. The LITAF domain is denoted by a coffee-colored triangle, while the low-complexity section is indicated in pink.

All *LITAF* genes were identified in the *H. armigera* genome, followed by analyses of gene collinearity and phylogeny. As shown in Figure 1A, cotton bollworm possesses 13 *HaLITAF* genes, located on chromosomes 1 (*HaLITAF1*), 5 (*HaLITAF2*, *HaLITAF3*, *HaLITAF4*), 10 (*HaLITAF5*, *HaLITAF6*, *HaLITAF7*, *HaLITAF8*) and 20 (*HaLITAF9*, *HaLITAF10*, *HaLITAF11*, *HaLITAF12*, *HaLITAF13*), respectively. Intragenomic collinearity analysis revealed no duplicated sequences among these 13 genes. Amino acid sequence consistency of 13 *Ha*LITAFs was 36.87%. Phylogenetic analysis grouped the *HaLITAFs* into two clusters: *HaLITAF1*, *HaLITAF6*, *HaLITAF3*, *HaLITAF10*, *HaLITAF9*, *HaLITAF4* and *HaLITAF2* in one cluster, and *HaLITAF7*, *HaLITAF8*, *HaLITAF12*, *HaLITAF11*, *HaLITAF13* and *HaLITAF5* in the other. All *Ha*LITAF proteins contain a C-terminal LITAF domain; additionally, all except *Ha*LITAF3, *Ha*LITAF5, *Ha*LITAF6 and *Ha*LITAF7 possess one to two low-complexity sections at the N-terminus (Figure 1B and Figure S1).

**Figure 2:**
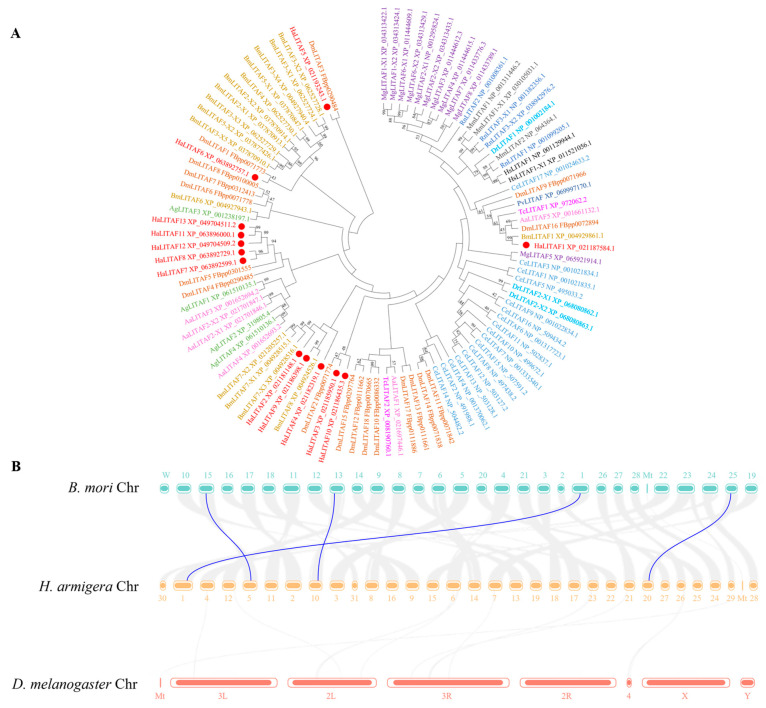
Phylogenetic analysis of the *HaLITAF* gene. (**A**) Phylogenetic tree depicting the LITAF of cotton bollworm alongside those from other species. HaLITAF, *Helicoverpa armigera*; BmLITAF, *Bombyx mori*; AaLITAF, *Aedes aegypti*; AgLITAF, *Anopheles gambiae*; DmLITAF, *Drosophila melanogaster*; TcLITAF, *Tribolium castaneum*; CeLITAF, *Caenorhabditis elegans*; MgLITAF, *Magallana gigas*; PvLITAF, *Penaeus vannamei*; DrLITAF, *Danio rerio*; MmLITAF, *Mus musculus*; RnLITAF, *Rattus norvegicus*; HsLITAF, *Homo sapiens*. (**B**) Diagram illustrating genome collinearity among *H. armigera*, *B. mori*, and *D. melanogaster*. Chromosomes are represented as colored rectangular segments. The grey lines connecting the segments denote the conservation of specific regions or genes across different chromosomes, with the *LITAF* gene highlighted by a blue line.

The neighbor-joining method was used to construct a phylogenetic tree of the *HaLITAF* genes alongside those from other species. As shown in Figure 2A, all *Ha*LITAF proteins cluster with insect LITAFs and are most closely related to the LITAFs of *B. mori* and *D. melanogaster*. A collinearity analysis was then performed on the genomes of *H. armigera*, *B. mori* and *D. melanogaster* (Figure 2B). This analysis revealed no duplication between the *LITAFs* of *H. armigera* and *D. melanogaster*. By contrast, repetitive sequences were detected between *H. armigera* and *B. mori LITAFs*, specifically *HaLITAF1* and *BmLITAF1*, *HaLITAF2* and *BmLITAF7-X2*, *HaLITAF5* and *BmLITAF2-X1*, and *HaLITAF9* and *BmLITAF8*.

### 3.2. Transcription Factor Analysis of HaLITAFs

**Figure 3:**
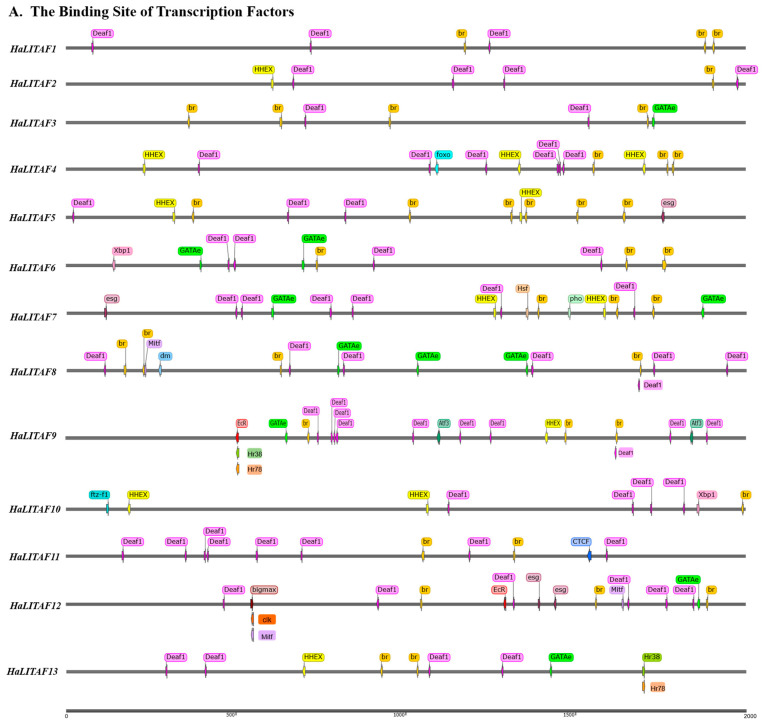

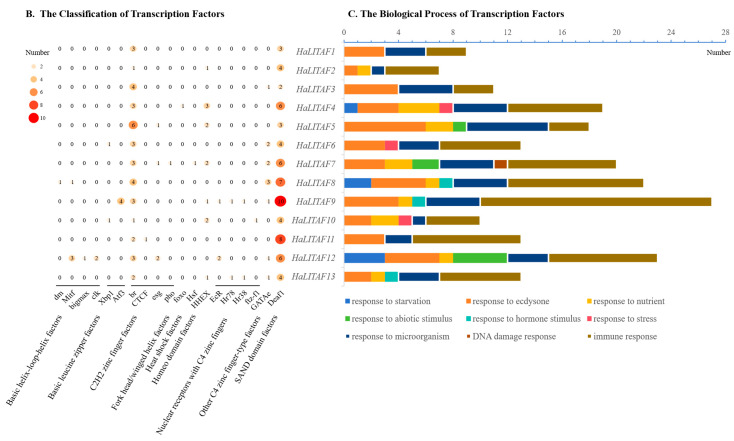
Analysis of cis-acting elements responsive to stimulus on the promoter of the *HaLITAF* gene. (**A**) A schematic diagram illustrating the binding sites of transcription factor (TF) on the promoter of the *HaLITAF* gene. The promoter fragment spans 2000 bp, with colored arrows indicating the length and strand orientation of the motif. (**B**) The classification of TF class associated with the *HaLITAF* gene. (**C**) The biological process classification of TF linked to the *HaLITAF* gene.

The upstream promoter sequences of 13 *HaLITAF* genes were retrieved from the cotton bollworm genome respectively, and the cis-acting elements responding to stimulus were analyzed. As shown in Figure 3A, the *HaLITAF1* promoter contained three Deaf1 and three br binding sites; *HaLITAF2* contained one HHEX, one br and four Deaf1 sites; *HaLITAF3* contained one GATAe, two Deaf1 and four br sites; *HaLITAF4* contained one foxo, three HHEX, three br and six Deaf1 sites; *HaLITAF5* contained one esg, two HHEX, three Deaf1 and six br sites; *HaLITAF6* contained one Xbp1, two GATAe, three br and four Deaf1 sites; *HaLITAF7* contained one esg, one Hsf, one pho, two HHEX, two GATAe, three br and six Deaf1 sites; *HaLITAF8* promoter contained one Mitf, one dm, three GATAe, four br and seven Deaf1 sites, *HaLITAF9* promoter contained one HHEX, one EcR, one Hr78, one Hr38, one GATAe, three br, four Atf3 and ten Deaf1 sites; *HaLITAF10* promoter contained one Xbp1, one br, one ftz—f1, two HHEX and four Deaf1 sites; *HaLITAF11* promoter contained one CTCF, two br and eight Deaf1 sites; *HaLITAF12* promoter contained one bigmax, one GATAe, two clk, two esg, two EcR, three Mitf, three br and six Deaf1 sites, and *HaLITAF13* promoter contained one HHEX, one Hr78, one Hr38, one GATAe, two br and four Deaf1 binding sites. Detailed descriptions of the relevant transcription factors are provided in the Supplementary Files, Table S1.

The transcription factors (TFs) of *HaLITAFs* in response to stimulus fall into nine classes, including basic helix–loop–helix factors (bHLH), basic leucine zipper factors (bZIP), C2H2 zinc finger factors, fork head/winged helix factors, heat shock factors, homeo domain factors, nuclear receptors with C4 zinc fingers, other C4 zinc-finger-type factors, and SAND domain factors. Of these, SAND domain factors exhibited the greatest number of TF binding sites, totaling 67. Functionally, the TFs of *HaLITAFs* were associated with nine biological processes: response to starvation, response to ecdysone, response to nutrient, response to abiotic stimulus, response to hormone stimulus, response to stress, response to microorganism, DNA damage response, and immune response. The immune response category contained the most TF binding sites, with a total of 87 (Figure 3B).

### 3.3. Differentially Expressed HaLITAF Under Short-Term Stress of 2-Tridecanone

**Figure 4:**
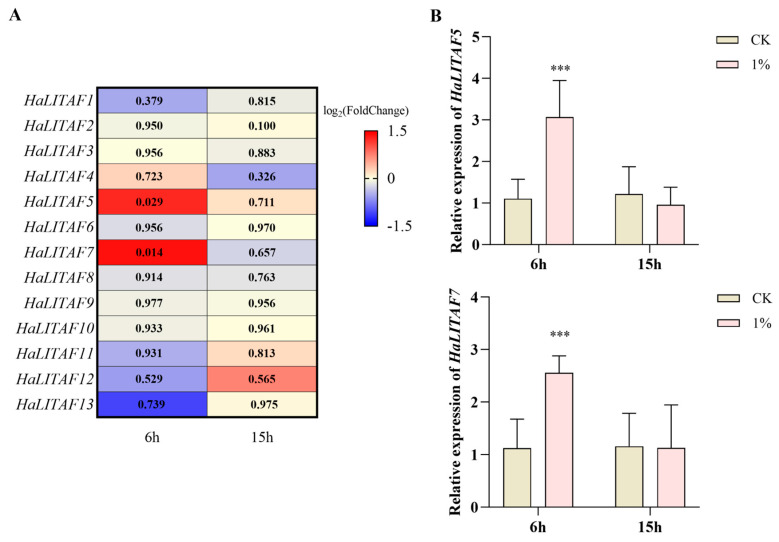
Analysis of differentially expressed genes of *HaLITAF* under short-term stress induced by 2-tridecanone. (**A**) Transcriptome analysis revealing the expression levels of *HaLITAF* following treatment with 2-tridecanone. The values represent *p*-values in every box. (**B**) qPCR validation of the relative expression levels of *HaLITAF5* and *HaLITAF7* subsequent to treatment with 2-tridecanone. Three asterisks “***”denote *p* < 0.001.

Using midgut transcriptome data following 2-tridecanone treatment, the differentially expressed *HaLITAF* genes were analyzed. The results in Figure 4A show that, after 6 h of exposure, the expression of *HaLITAF1*, *HaLITAF2*, *HaLITAF3*, *HaLITAF4*, *HaLITAF6*, *HaLITAF8*, *HaLITAF9*, *HaLITAF10*, *HaLITAF11 and HaLITAF12* remained essentially unchanged. By contrast, *HaLITAF5* (1.241, *p* = 0.0288) and *HaLITAF7* (1.363, *p* = 0.014) were significantly upregulated, while *HaLITAF13* was downregulated but not significantly (−1.092, *p* = 0.739). At 15 h post-treatment, none of the *HaLITAF* genes showed significant change. The qPCR validation confirmed that *HaLITAF5* and *HaLITAF7* were significantly upregulated after 6 h of 2-tridecanone exposure (Figure 4B).

### 3.4. Sequence Analysis of HaLITAF5 and HaLITAF7

The mRNA sequences of *HaLITAF5* and *HaLITAF7* were isolated from the midgut tissues of sixth-instar larvae. The coding sequence (CDS) of *HaLITAF5* was 237 bp, encoding 78 amino acids, with a predicted protein molecular mass of 8.68 kD and an isoelectric point of 8.37. The CDS of *HaLITAF7* was 342 bp, encoding 113 amino acids, with a predicted molecular mass and isoelectric point of 12.26 kD and 6.23, respectively. The amino acid sequence similarity between *Ha*LITAF5 and *Ha*LITAF7 was 44.25%. Notably, both sequences feature the conserved CXXC motif (CPSC, positions 15–18 in *Ha*LITAF5 and positions 50–53 in *Ha*LITAF7) and the HXCPXCXXXXG motif (HYCPNCSSYLG at positions 64–74 in *Ha*LITAF5 and HYCPNCSAYLG at positions 99–109 in *Ha*LITAF7), placing them within the zf-LITAF-like superfamily (Figure S2).

**Figure 5:**
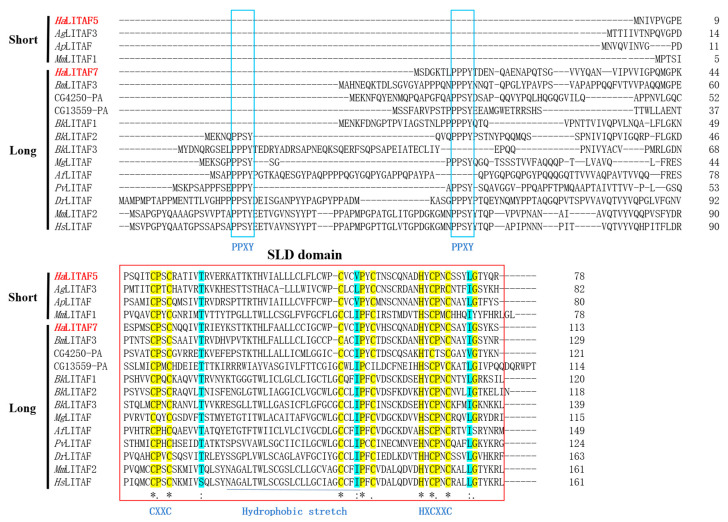
Multiple sequence alignment of *Ha*LITAF5 and *Ha*LITAF7 with LITAFs from other species that reported to have immune functions in the literature. The two motifs (CXXC and HXCXXC) are highlighted in blue capitals. The PPXY motif located in the N-terminus is shown by blue boxes. The hydrophobic stretch is marked by a blue line. The SLD domain is identified by a red box, in which the “*”, “.”, “:” denote completely conserved, partially similar and homogeneously substituted amino acids, respectively. *Ha*LITAF5 and *Ha*LITAF7, *Helicoverpa armigera*, XP_021193243.1 and XP_063892599.1; *Bm*LITAF3, *Bombyx mori*, XP_062527730.1; *Ap*LITAF, *Antheraea pernyi*, GWHGABGR005336; *Ag*LITAF3, *Anopheles gambiae*, XP_319805.4; CG13559-PA, CG4250-PA and CG4250-PB, *Drosophila melanogaster* LITAF, FBpp0071966 and FBpp0071776; *Bk*LITAF1, *Bk*LITAF2 and *Bk*LITAF3, *Brachionus koreanus*, ALG36745.1, ALG36746.1 and ALG36747.1; *Mg*LITAF, *Magallana gigas*, ABO70331.1; *Af*LITAF, *Azumapecten farreri*, ABI79459.1; *Pv*LITAF, *Penaeus vannamei*, AEK86526.1; *Dr*LITAF, *Danio rerio*, NP_001002184.1; *Mm*LITAF1 and *Mm*LITAF2, *Mus musculus*, NP_001311446.2 and NP_064364.1; *Hs*LITAF1 and *Hs*LITAF2, *Homo sapiens*, NP_001129944.1 and NP_001129945.1.

The amino acid sequences of *Ha*LITAF5 and *Ha*LITAF7 were aligned with the published LITAF proteins associated with the immune response. Figure 5 indicates that all LITAF proteins contain a LITAF domain, also referred to as the SIMPLE-like domain (SLD). This domain comprises a conserved CXXC motif and an HXCXXC motif, which are separated by a hydrophobic stretch. *Ha*LITAF7, a long-chain LITAF, features a typical PPXY motif at the N-terminus, whereas *Ha*LITAF5, a short-chain LITAF, lacks the PPXY motif at the N-terminus.

The predicted tertiary structure of *Ha*LITAF5 consists of one helix (T32-L45) and five sheets (S11-C15, A20-T24, R25-T31, C59-C66, S71-Y76), lacking disulfide bonds and signal peptides. It is likely to be attached to the outer nuclear membrane or the outer lysosomal membrane through the transmembrane segment spanning T34-T56. *Ha*LITAF7 comprises two helices (T67-I79, C86-S93) and five sheets (P47-S49, Q56-V58, R60-S65, Q95-Y100, Y107-Y111), without disulfide bonds, signal peptides, and transmembrane segments, and it maybe resides in the cytoplasm (Figure S3).

### 3.5. Spatiotemporal Expression Analysis of HaLITAF5 and HaLITAF7

**Figure 6:**
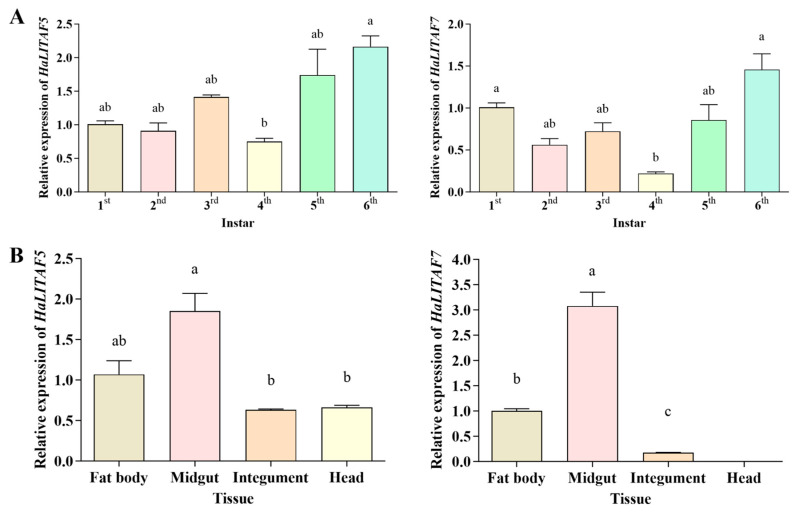
Relative expression levels of *HaLITAF5* and *HaLITAF7* in different larval stages (**A**) and different tissues of sixth instar (**B**). Note: Different lowercase letters indicate significant differences (*p* < 0.05).

The expression profiles of *HaLITAF5* and *HaLITAF7* were tested in different larval instars and tissues (Figure 6). Both *HaLITAF5* and *HaLITAF7* were expressed in all larval instars, reaching their lowest levels during the fourth instar (0.75 and 0.22) and peaking in the sixth instar (2.16 and 1.46), with a notable disparity between these two stages. *HaLITAF5* was expressed in the fat body, midgut, integument, and head of the sixth-instar larvae, with the highest expression detected in the midgut (1.85), significantly surpassing levels in the integument and head. Similarly, *HaLITAF7* was found in the fat body, midgut, and integument of sixth-instar larvae, with the most pronounced expression observed in the midgut (3.08) while being barely detectable in the head.

### 3.6. Expression Analysis of HaLITAF5 and HaLITAF7 After Plant Secondary Metabolite Stress

Sixth-instar larvae with varying concentrations of plant secondary metabolites were examined, and the intestinal relative expression levels of *HaLITAF5* and *HaLITAF7* were assessed using qPCR.

**Figure 7:**
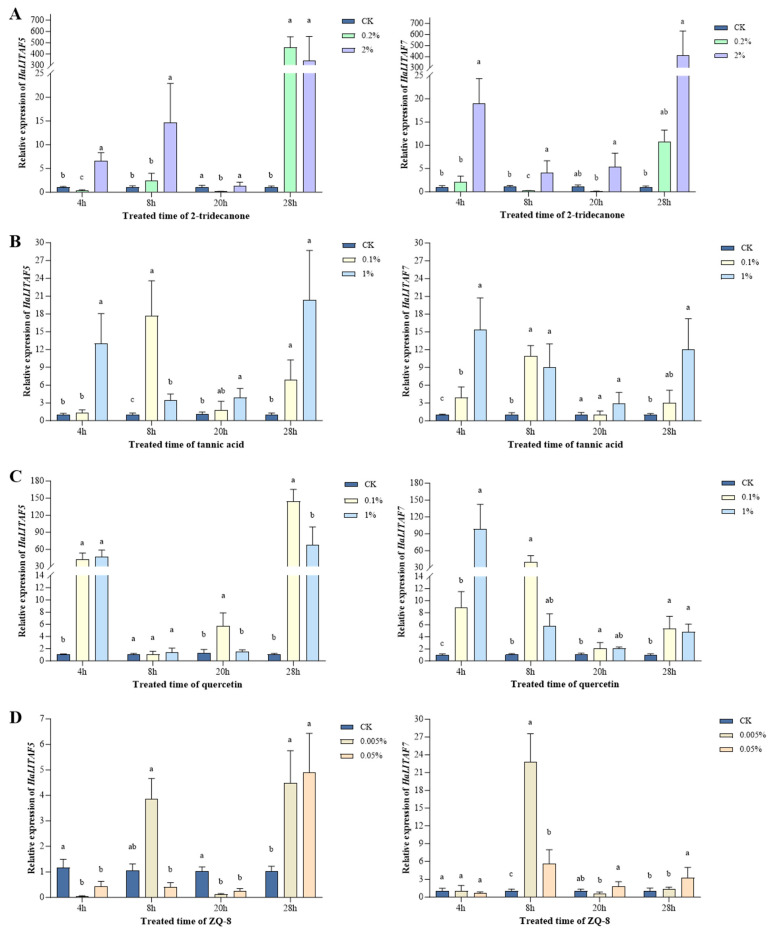
Relative expression levels of *HaLITAF5* and *HaLITAF7* in different plant secondary metabolites exposed to sixth-instar larvae, respectively: (**A**) 2-tridecanone, (**B**) tannic acid, (**C**) quercetin and (**D**) ZQ-8. Note: Different lowercase letters indicate significant differences (*p* < 0.05).

Both the concentration of 2-tridecanone and the duration of stress influence the expression levels of *HaLITAF5* (F_df2_ = 9.65, *p* = 0.0008 for concentration; F_df3_ = 34.77, *p* < 0.0001 for time). Following treatment with a high concentration (2%) for 4 h, the expression level of *HaLITAF5* was significantly elevated compared to the control group (*p* < 0.0001). Similarly, after treatment with a low concentration (0.2%) for 28 h, the expression level of *HaLITAF5* also showed a significant increase relative to the control group (*p* = 0.0004). Furthermore, both the concentration of 2-tridecanone and the duration of stress similarly affected the expression levels of *HaLITAF7* (F_df2_ = 11.71, *p* = 0.0003 for concentration; F_df3_ = 10.7, *p* = 0.0001 for time). Treatment with a high concentration (2%) for 4 h resulted in the expression level of *HaLITAF7* being 17.92 times greater than that of the control group (*p* < 0.0001) (Figure 7A).

The expression level of *HaLITAF5* was 11.98 times greater than that of the control after 4 h of exposure to high-concentration (1%) tannic acid (*p* = 0.0056). Following 8 h of treatment with low-concentration (0.1%) tannic acid, the expression level of *HaLITAF5* increased to 16.68 times that of the control (*p* = 0.008). Both the concentration of tannic acid and the duration of stress significantly influenced the expression level of *HaLITAF5* (F_df2_ = 20.93, *p* < 0.0001 for concentration; F_df3_ = 6.97, *p* = 0.0016 for time). Similarly, the concentration and duration of tannic acid treatment also had a notable impact on the expression level of *HaLITAF7* (F_df2_ = 31.93, *p* < 0.0001 for concentration; F_df3_ = 7.40, *p* = 0.0011 for time). After 4 h of treatment, both low-concentration (0.1%) and high-concentration (1%) tannic acid significantly elevated the expression level of *HaLITAF7*, with increases of 2.19 and 14.32 times that of the control, respectively (*p* = 0.007 for 0.1%, *p* = 0.0004 for 1%) (Figure 7B).

Both low (0.1%) and high (1%) concentrations of quercetin markedly enhanced the expression of *HaLITAF5* compared to the control following 4 h of stress treatment, showing a 40.93-fold increase (*p* = 0.0019) and a 45.82-fold increase (*p* = 0.0011) relative to the control, respectively. The expression of *HaLITAF5* was significantly influenced by both the concentration of quercetin used for treatment and the duration of stress exposure (F_df2_ = 15.51, *p* < 0.0001 for concentration; F_df3_ = 21.91, *p* < 0.0001 for time). Similarly, both low (0.1%) and high (1%) concentrations of quercetin elevated the expression of *HaLITAF7* compared to the control after 4 h of treatment, resulting in a 7.89-fold increase (*p* = 0.0099) and a 97.29-fold increase (*p* = 0.0138) relative to the control, respectively. The expression of *HaLITAF7* was significantly impacted by both the concentration of quercetin used for treatment and the duration of stress exposure (F_df2_ = 4.162, *p* = 0.0281 for concentration; F_df3_ = 4.354, *p* = 0.0139 for time) (Figure 7C).

The expression levels of *HaLITAF5* were significantly decreased following 4 h of stress treatment with the ferulic extract analogue ZQ-8 at both low and high concentration (*p* = 0.0029 for 0.005%, *p* = 0.0367 for 0.05%). Subsequently, after 28 h of treatment, the expression levels of *HaLITAF5* significantly increased (*p* = 0.0178 for 0.005%, *p* = 0.0076 for 0.05%). The duration of stress induced by ZQ-8 significantly influenced the expression level of *HaLITAF5* (F_df3_ = 14.24, *p* < 0.0001 for time). Following an 8-h treatment with ZQ-8 at concentrations of 0.005% and 0.05%, the expression levels of *HaLITAF7* markedly rose, showing a 21.8-fold increase (*p* < 0.0001) and a 4.589-fold increase (*p* = 0.0026) compared to the control, respectively. Both the concentration of treatment and the duration of stress caused by ZQ-8 had a notable impact on the expression level of *HaLITAF7* (F_df2_ = 32.85, *p* < 0.0001 for concentration; F_df3_ = 60.04, *p* < 0.0001 for time) (Figure 7D).

The results presented above suggest that following treatment with 2-tridecanone, tannic acid, quercetin, and ZQ-8, the relative expression levels of *HaLITAF5* and *HaLITAF7* increased. However, the trends in their expression changes were not identical. This observation leads to the speculation that *HaLITAF5* and *HaLITAF7* may serve distinct roles in the response to stress induced by various secondary metabolites.

## 4. Discussion

Plant secondary metabolites are synthesized to provide defensive functions and regulate defense signaling pathways to safeguard plants against herbivores [32]. The cotton bollworm can feed on a variety of host plants, indicating that it also has a powerful detoxification and metabolic system. These systems enable the larvae to mitigate the detrimental effects of plant secondary metabolites while simultaneously extracting essential nutrients from the plants [33]. Furthermore, the adaptive mechanisms employed by the cotton bollworm in response to plant secondary metabolites were intricately linked to the development of its resistance to pesticides [34].

This study employed the transcriptome after short-term stress induced by 2-tridecanone to identify two markedly upregulated genes from the *Ha*LITAF family, specifically *HaLITAF5* and *HaLITAF7* (see Figure 4). Furthermore, when the larvae were exposed to four distinct types of plant secondary metabolites, namely 2-tridecanone, tannic acid, quercetin, and ZQ-8, the expression levels of *HaLITAF5* and *HaLITAF7* in their midgut increased markedly (see Figure 7). 2-Tridecanone, a volatile methyl ketone compound serving as both a phyto-pheromone and natural insecticide, represents an important secondary metabolite in wild tomatoes [35]. Studies have shown that 2-tridecanone induces overexpression of various detoxification enzymes in cotton bollworm and increases larval resistance to deltamethrin [36]. Tannic acid, a natural polyphenol crude extract compound, exists in plant vascular tissues [37]. High concentrations of tannic acid could reduce the digestive efficiency and protease activity in the midgut, thereby inhibiting the larval growth of cotton bollworm [38]. Quercetin belongs to the class of flavonoids and is found in a variety of vegetables, fruits and spices. It could increase the activity of multiple detoxification enzymes in cotton bollworm [39,40]. ZQ-8, an artificial analog of Tschimganin 146 isolated from ferula, belongs to terpenoid ester compounds [41]. Yang et al. showed that after treating the third-instar larvae of cotton bollworm with ZQ-8, the larval molting was blocked, the pupation duration was prolonged, the pupation and eclosion rates were significantly reduced, and even deformed pupae were formed [42]. Consequently, the overexpression of *HaLITAF5* and *HaLITAF7* induced by plant secondary metabolites may play a role in the detoxification metabolism and growth and development of *H. armigera*.

The class Insecta represents the largest group within the animal kingdom; however, research on insect LITAF remains limited. In *D. melanogaster* infected with bacteria, two *LITAF* genes are notably upregulated in hemocytes [28]. Similarly, in the intestine of *A. gambiae* infected with plasmodium, *LITAF-like 3* (*LL3*) is upregulated, while *LITAF-like 6* (*LL6*) is specifically expressed in hemocytes [29]. Furthermore, in the intestine of *Antheraea pernyi* infected with nucleopolyhedrovirus, *Escherichia coli*, and *Bacillus subtilis*, a significant upregulation of *ApLITAF* expression is observed [43]. Collectively, these findings suggest that insect LITAF plays a crucial role in responding to the stress induced by exogenous pathogens and is involved in the immune response of insects.

This study primarily investigated the binding sites for 19 transcription factors with response to stimulus in the promoters of each *HaLITAF* gene. The findings revealed the presence of cis-acting elements for Deformed epidermal autoregulatory factor 1 (Deaf1) and Broad-complex proteins (br) in all 13 *HaLITAF* genes (see Figure 3). Deaf1 is involved in the regulation of the immune response, and br can respond to ecdysone and respond to symbiont [44,45,46]. Shiomi et al. identified a 1.2 kb promoter region of human *HsLITAF* and found that a 34 bp nucleotide sequence from −74 to −43 within this promoter displayed the highest transcriptional activity. This region was predicted to harbor a binding site for hepatocyte nuclear factor (HNF-3α), which contains a forkhead/winged helix domain as the DNA binding domain and regulates the transcription of many kinds of genes [47]. There was only one binding site of the forkhead box O protein (foxo) in the promoter of *HaLITAF4* (see Figure 3).

The N-terminus of *Hs*LITAF contains two PPXY motifs that mediate protein–protein interactions with WW-domain-containing proteins, including WWOX, Nedd4, TSG101, Itch, etc. [12]. In the *Ha*LITAF family, the predicted three proteins do not contain the PPPY motif, which are *Ha*LITAF3, *Ha*LITAF4, and *Ha*LITAF5 (see Figure S1). Additionally, multiple sequence alignment results indicate that *Ha*LITAF5 lacks the PPPY motif, whereas *Ha*LITAF7 possesses one PPPY motif (see Figure 5). Shi et al. identified that five human genes with a PPXY motif could enhance glutathione and endocytotic marker uptake in yeast, including LITAF, thereby impacting the plasma membrane transport activity [48]. The homologs of the *LITAF* gene were encoded by some aquatic animal viruses. The amino acid sequences of these LITAF homologs were relatively short and lacked the PPPY motif [49]. These viral LITAF homologs could interact with the host’s original LITAF, prompting its relocation from late endosomes/lysosomes to early endosomes. Intriguingly, altering the two PPXY motifs of the native LITAF does not impact its co-localization with viral LITAF homologs [50,51]. While both *HaLITAF5* and *HaLITAF7* could be overexpressed in response to plant secondary metabolites, their distinct roles remain unclear in *H. armigera*.

Additionally, this study examined the expression levels of *HaLITAF5* and *HaLITAF7* during the larval stages of *H. armigera*. The results revealed that both genes were highly expressed in sixth-instar larvae, particularly in the midgut (see Figure 6). The cotton bollworm completes three generations per year in the Xinjiang region of China, with the second and third generations causing more severe damage. Among these two generations, the fifth- and sixth-instar larvae exhibit the highest damaging capacity [52]. As larger larvae consume substantial amounts of plant material, they also ingest considerable quantities of plant secondary metabolites. The findings presented in Figure 6 provide an additional perspective, suggesting that *HaLITAF5* and *HaLITAF7* may be involved in the detoxification and metabolism of plant secondary metabolites in *H. armigera*. Similarly, *LITAF* expression demonstrated strong tissue specificity across different tissues of *A. pernyi*, with expression restricted to the gut of fifth-instar larvae. The authors speculate that the specific expression of *ApLITAF* in the gut was due to the primary route of pathogen invasion and indicated a potential role in biotic stress responses in *A. pernyi* [43].

This study explored the response of *LITAF* to spatial, temporal, and plant secondary metabolite stresses in *H. armigera*. Our findings revealed the upregulation of two crucial genes, *HaLITAF5* and *HaLITAF7*, following exposure to plant secondary metabolites, indicating their potential involvement in detoxification processes in cotton bollworms. Subsequently, techniques such as RNA interference, verification of promoter transcriptional activity, and yeast hybridization will be employed to investigate the functionality of these *HaLITAF* genes. This research aims to elucidate the precise contributions of LITAF to the growth, development, immune response, and detoxification metabolism of cotton bollworm, with the ultimate goal of pinpointing a novel, eco-friendly, and scientifically sound target for managing *H. armigera*.

## 5. Conclusions

The cotton bollworm is an extremely destructive agricultural pest that can pose a serious threat to a variety of crops. Its genome comprises 13 *LITAF* genes, which could be involved in various biological processes associated with response to stimulus. Notably, two overexpressed genes, *HaLITAF5* and *HaLITAF7*, have been identified as candidate regulators in response to the stress by various plant secondary metabolites. This study establishes a foundation for further investigation into the roles of these genes in response to plant secondary metabolite and insecticide stresses. Additionally, it offers new target sites for managing insecticide resistance in cotton bollworm.

## Figures and Tables

**Table 1:** The primers used in this study.

Primers	Sequence (5′ to 3′)	Purpose
*HaLITAF5*-5O	TCCCTCCATCTCAAGTGTTCTGTATG	5′RACE PCR
*HaLITAF5*-5I	GGCAGTAGTGGTCGGCGTTCTG	
*HaLITAF7*-5O	GTTGCAGGATGGGCAGGACA	
*HaLITAF7*-5I	GAAGGGTTTTGCCGTCGCTC	
*HaLITAF5*-3O	AAAGCCACCACCAAGACTCATGT	3′RACE PCR
*HaLITAF5*-3I	TGCTGTTGTGTTTGTTCCTTTGTT	
*HaLITAF7*-3I	ATGAGCGACGGCAAAACCC	
*HaLITAF7*-3O	CATTGGCAGTTACAAGAGCTAG	
*HaLITAF5*-F	GGAATTCATGAATATCGTTCCCGTTGG	CDS PCR
*HaLITAF5*-R	CCAAGCTTCTATCGTTGGTACGTTCCT	
*HaLITAF7*-F	GGAATTCATGAGCGACGGCAAAACCCT	
*HaLITAF7*-R	CCAAGCTTCTAGCTCTTGTAACTGCCA	
*HaLITAF5*-QF	GAACCTTCCCAGATCACG	qPCR
*HaLITAF5*-QR	ACAAAGGAACAAACACAAG	
*HaLITAF7*-QF	ACAGATGAGAACCAGGCG	
*HaLITAF7*-QR	TTTGGCAACTGTGGACGC	
*GADPH*-QF	CCAGAAGACAGTGGATGGAC	
*GADPH*-QR	TACCAGTCAGCTTTCCGTTC	
*RPS3*-QF	ACGGAGTTTTCAAGGCGGAA	
*RPS3*-QR	GACTGCTCCGGGATGTTGAA	
*RPL27*-QF	ACAGGTATCCCCGCAAAGTGC	
*RPL27*-QR	GTCCTTGGCGCTGAACTTCTC	
*RPL32*-QF	CATCAATCGGATCGCTATG	
*RPL32*-QR	CCATTGGGTAGCATGTGAC	
*RPS15*-QF	CCGAGATTGTTAAGACAC	
*RPS15*-QR	GTATGTGACTGAGAACTC	

## Data Availability

The original data are included in this article; further inquiries can be directed to the corresponding authors.

## Supplementary Materials

The following supporting information can be downloaded at: https://www.mdpi.com/article/10.3390/biology15080595/s1, Table S1: The Excel data of cis-acting elements responsive to stimulus on the promoter of the *HaITAF* gene; Figure S1: The amino acid sequence alignment of 13 LITAFs in *H. armigera*. The two key motifs of the LITAF domain are marked in yellow, and the PPXY motif at the N-terminus is marked in blue; Figure S2: The nucleotides and amino acids of *HaLITAF5* and *HaLITAF7*. The underlines mark the two motifs, CXXC and HXCPXCXXXXG. The red and blue letters represent the start codon and stop codon respectively; Figure S3: The predicted secondary and tertiary structures of *Ha*LITAF5 (A) and *Ha*LITAF7 (B). In secondary structure, helices label H1 and H2, strands are shown by their sheets A and B, β shows a beta turn, γ shows a gamma turn, red paper clip represents a beta hairpin. In the tertiary structure, the N terminus of the protein is in dark blue, while the C terminus is in red.
