# Supplementary figures and images for: Identification and Screening of LITAF Family Key Genes Responsive to Plant Secondary Metabolites in Helicoverpa armigera
Source: Biology (Basel). 2026 Apr 9;15(8):595. doi: 10.3390/biology15080595 (PMC13113599; doi:10.3390/biology15080595)

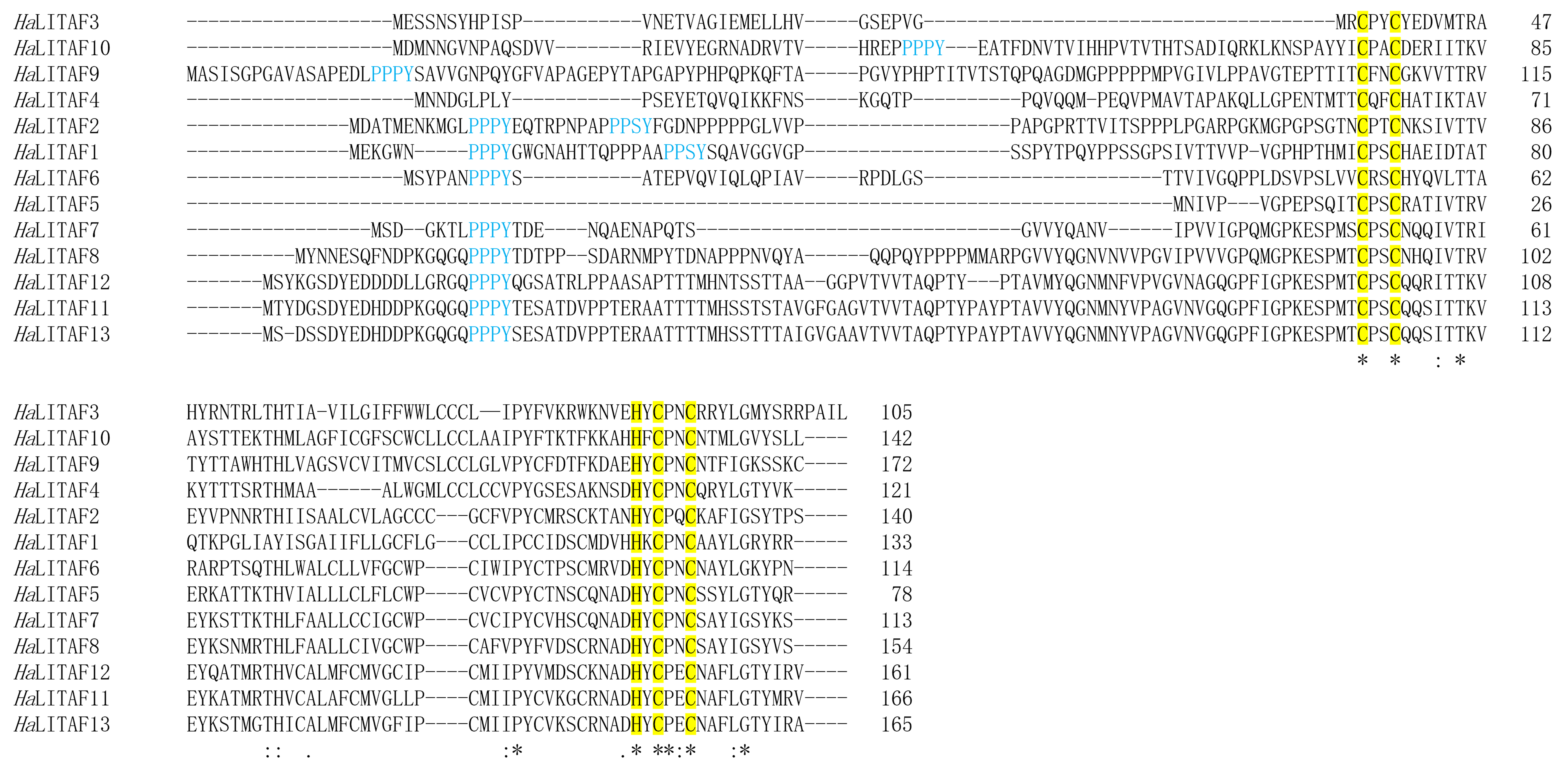

Supplement: Supplementary file 1 [file biology-15-00595-s001.zip › Supplementary files/Figure S1.tif]

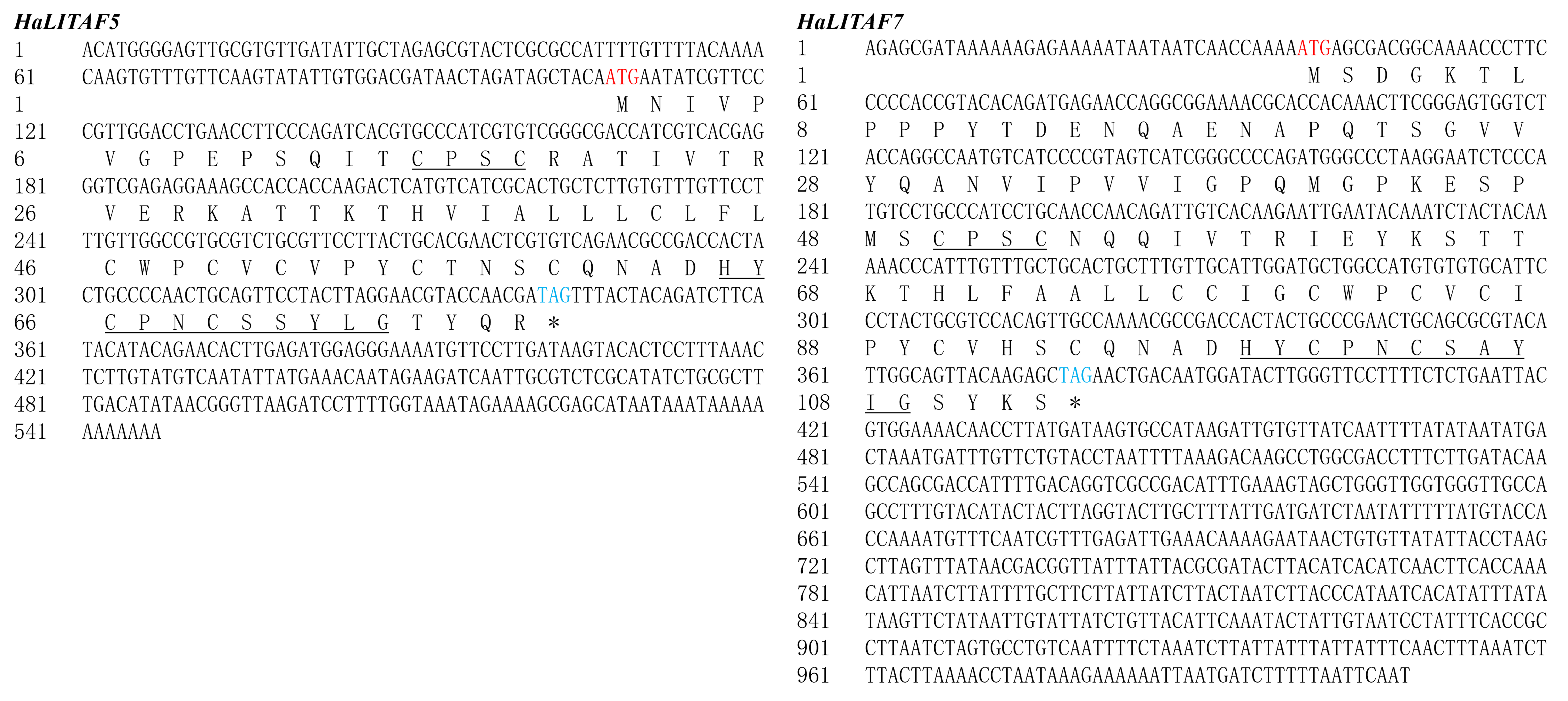

Supplement: Supplementary file 1 [file biology-15-00595-s001.zip › Supplementary files/Figure S2.tif]

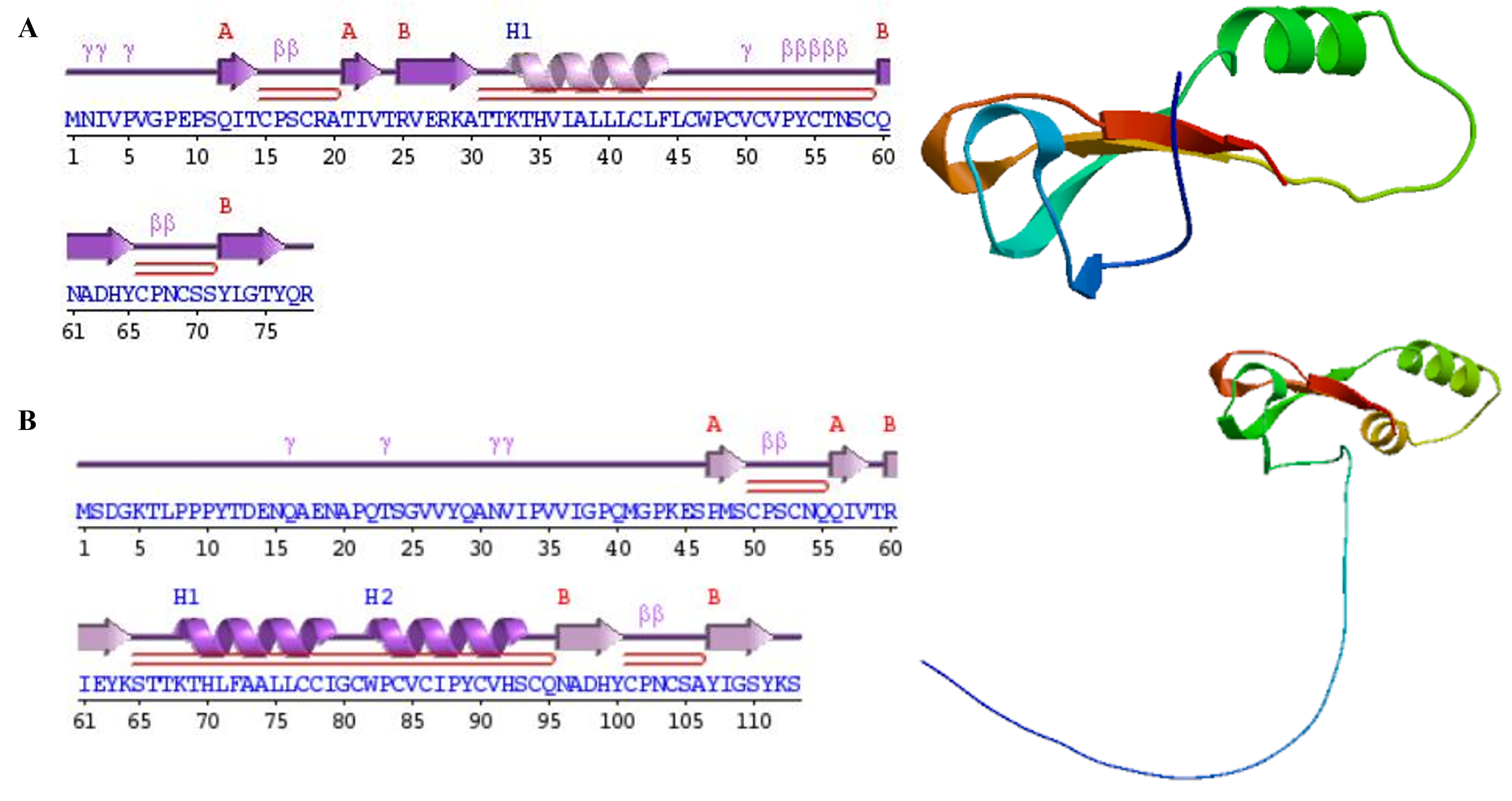

Supplement: Supplementary file 1 [file biology-15-00595-s001.zip › Supplementary files/Figure S3.tif]
